# *Bacillus coagulans* regulates gut microbiota and ameliorates the alcoholic-associated liver disease in mice

**DOI:** 10.3389/fmicb.2024.1337185

**Published:** 2024-02-26

**Authors:** Zhenzhen Liu, Tong Liu, Zhenting Zhang, Yurong Fan

**Affiliations:** ^1^Antibiotics Research and Re-evaluation Key Laboratory of Sichuan Province, Sichuan Industrial Institute of Antibiotics, School of Pharmacy, Chengdu University, Chengdu, China; ^2^State Key Laboratory of Agricultural Microbiology, Hubei Hongshan Laboratory, College of Life Science and Technology, Huazhong Agricultural University, Wuhan, China; ^3^The Key Laboratory of Environmental Pollution Monitoring and Disease Control, Ministry of Education, School of Public Health, Guizhou Medical University, Guiyang, China

**Keywords:** *Bacillus coagulans*, alcoholic-associated liver diseases, microbiota, short-chain fatty acids, intestinal barrier

## Abstract

**Introduction:**

Alcoholic-associated liver diseases (ALD) are now widespread issues worldwide. Alcoholic-induced chronic dysbiosis of the gut microbiota is one of the factors in the pathophysiology of ALD.

**Methods:**

In this work, we employed a chronic-binge ethanol feeding mice model, as described in a previous report.

**Results:**

Our findings demonstrate that hepatic inflammatory injury damage and accumulation of fat can be effectively reduced in mice with ALD by altering the gut microbiota utilizing *Bacillus coagulans*. Treatment with *B. coagulans* significantly modulates the levels of TNF-α, IL-1β, and IL-22 cytokines while maintaining tight junction proteins and mucin protein expressions to support intestinal barrier function restoration. Treatment with *B. coagulans* also alters the composition of the gut microbiota and increases the production of short-chain fatty acids (SCFAs).

**Discussion:**

This is mostly due to *B. coagulans* promotes the growth of bacteria that produce SCFAs, such as *Ruminococcus* species and *Akkermansia*, while inhibiting the growth of pathogenic bacteria like *Escherichia Shigella*. Moreover, treatment with *B. coagulans* causes levels of *2-Ketobutyric acid*, *ketoleucine*, and *indoleacetic acid* increase while *homovanillic acid* and *3’-O-Methylguanosine* metabolites decrease significantly. This study facilitates the development of therapeutic and preventive strategies for ALD using lactic acid bacteria.

## Introduction

Chronic alcohol abuse is one of the most common causes of death from liver disease (GBD 2016 Alcohol Collaborators, 2018). According to estimates from the World Health Organization’s “Report on the Word Alcohol and Health Situation,” 5.1% of the global illness burden in 2016 was attributable to alcohol-related deaths, which totaled close to 3 million cases (WHO, 2018). Chronic liver damage, inflammation, and fibrosis are all possible outcomes of alcoholic-associated liver disease (ALD). Moreover, it may result in alcoholic steatohepatitis (ASH), which can then progress to hepatocellular carcinoma (HCC), which may be deadly (Seitz et al., 2018; Lackner and Tiniakos, 2019). Since ALD has become more common each year, it is imperative to find an effective treatment for the condition.

Alcohol-induced oxidative stress and inflammation are the main causes of steatohepatitis and liver damage (Ceni et al., 2014; Gao et al., 2019; Seitz, 2020). The cornerstones of ALD therapy these days are phosphodiesterase inhibitors, glucocorticoids, polyene phosphatidylcholine, and other medications (Singal et al., 2018). However, continued use of these medications often results in drug resistance and increases the risk of infection. Only a few kinds of drugs, including acamprosate, disulfiram and naltrexone, have gained Food and Drug Administration (FDA) approval for therapy of ALD patients to yet (Coe et al., 2023). Moreover, it is without dispute that *Enterococcus faecalis* produces cytolysins that contribute to the progression of ALD. In mice with ALD models, phage therapy has also been shown to reduce liver damage (Llorente et al., 2017; Duan et al., 2019), however, phages’ limited host specificity restricts their use.

A family of living microorganisms known as probiotics can directly and dose-wise improve the gut microbiota’s equilibrium (Neish, 2009). Probiotics regulate gut microbiota and repair intestinal barrier dysfunction caused by alcohol by reducing intestinal mucosal permeability and preventing intestinal bacterial translocation (Grander et al., 2018; Gu et al., 2019). Probiotics are thus unquestionably an innovative, simple-to-use treatment alternative with no unfavorable side effects for alcoholic-associated liver disease. It has been demonstrated that ALD animals and patients with ALD without cirrhosis had lower levels of *Firmicute* (Sun et al., 2020).

Notably, a variety of *Bacillus* spp. strains have been utilized as probiotic-containing dietary supplements (Mingmongkolchai and Panbangred, 2018). Compared to other probiotics, it has a higher acid resistance and maintains its stability when heated and stored at low temperatures (Elshaghabee et al., 2017). *Bacillus coagulans* not only inhibits viruses but also regulates the immune system and promotes the growth of beneficial gut microbes (Hao et al., 2015). Additionally, *B. coagulans* has immunomodulatory effects by inhibiting the inflammatory cytokine IL-8 production, increasing the secretion of the pro-inflammatory cytokine IL-10, and reversing lipopolysaccharide-induced inflammation (Shinde et al., 2019). These findings suggest that *B. coagulans* reduces inflammatory-induced tissue damage and supports the host’s immune system’s defense against infection.

In this work, we employed a chronic-binge ethanol feeding mice model, as described in a previous report (Bertola et al., 2013) in this work. We investigated *B. coagulans’* potential therapeutic value for ALD. Our study’s findings, which include reduced liver histopathologic damage, enhanced host immunity, and regulated gut microbiota, show that *B. coagulans* supplementation can mitigate liver injury in ALD.

## Materials and methods

### Mice ethanol feeding and treatments

Huazhong Agricultural University’s Institutional Animal Care and Use Committee regulations (HZAUMO-2021-0010) were followed when caring for the animals. Purchased and used in Huazhong Agricultural University’s Laboratory Animal Center were C57BL/6 male mice (7–8 weeks old, average weight of 20 g). According to Bertola et al., mice were fed a 10-day Lieber-DeCarli diet that contained ethanol (Bertola et al., 2013).

**Figure 1:**
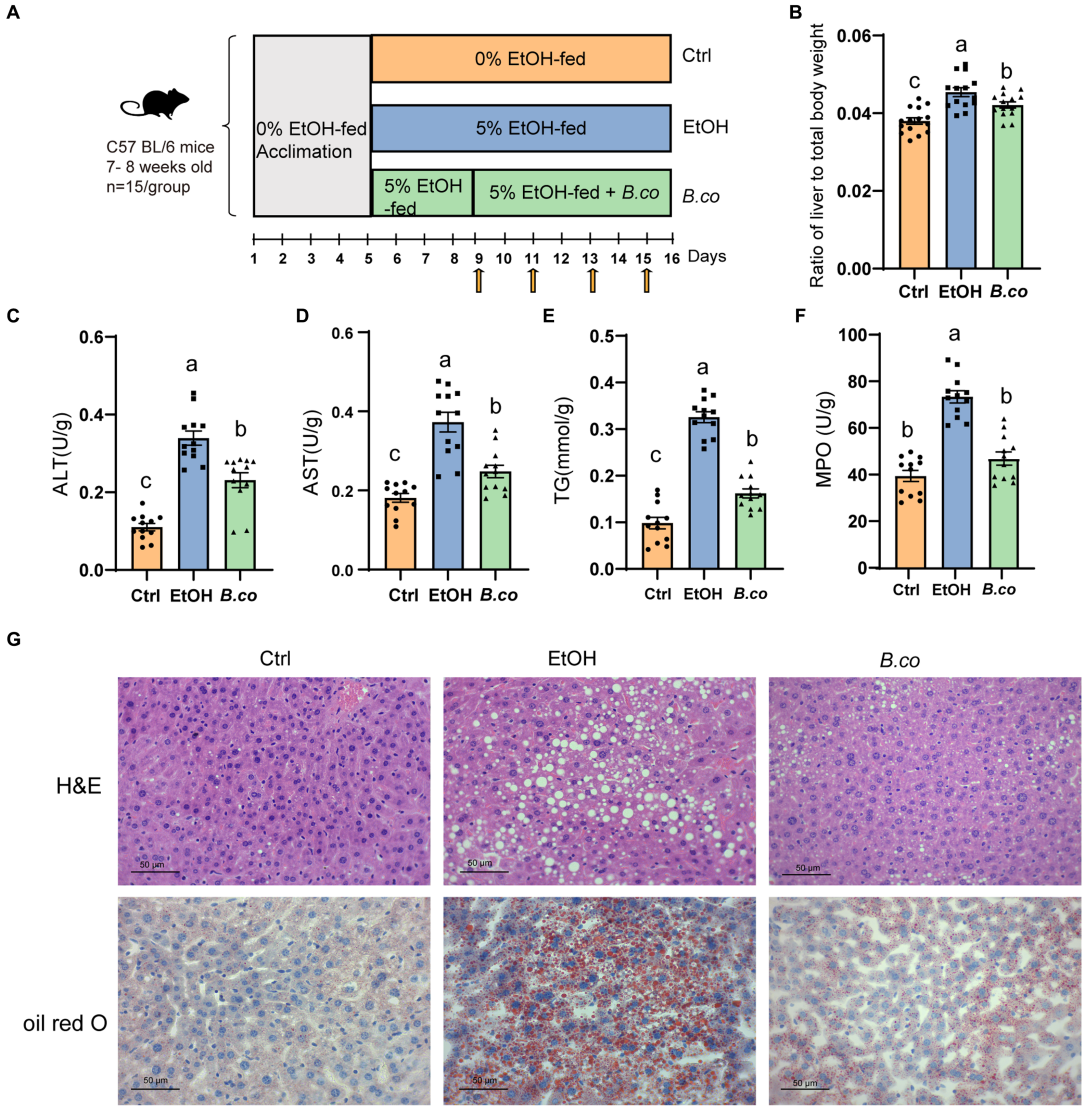
*B. coagulans* reduced histological damage of liver induced by ethanol. **(A)** Diagram illustrating the mouse model of ethanol-induced ALD employed in this study. Arrows indicate gavage phosphate bufer saline (PBS) or *B. coagulans* treatments. **(B)** Liver-to-body-weight ratio. **(C)** Serum ALT concentration. **(D)** Serum AST concentration. **(E)** Liver triglyceride concentration. **(F)** Liver myeloperoxidase activity. **(G)** Histological assessment of steatosis with representative pictures of H&E staining (up) and oil-red-O stained (down) liver sections. Scale bars represent 50 μm. The concentrations of panels **(C,D,F)** were calculated as follows: Activity calculated using the kit (U/L)/ total protein concentration (g/L). Data are shown as means ± SEM. Data with different superscript letters (a, b, and c) are significantly different (*p* < 0.05) according to one-way ANOVA followed by Tukey’s test. Ctrl, healthy control group; EtOH, ethanol-induced group; *B. co*, supplementation of *B. coagulans* group.

The Lieber-DeCarli pair-fed diet was given to all mice for 5 days in order to adapt them to a liquid diet. The mice in the therapy group and ALD model group were subsequently given the Lieber-DeCarli ethanol diet containing 5% ethanol. The ethanol-fed mice were calorie-matched to the animals in the healthy control group. In our study, starting on the ninth day, a subgroup of ethanol-fed mice was given a supplement of *Bacillus coagulans* (1.0 × 10^9^ CFU) by gavage every 2 days as a therapeutic measure. An equivalent volume of PBS was gavaged to the control groups. The ALD model group and treatment group gavaged ethanol (5 g kg-1 body weight) and the healthy control group gavaged isocaloric maltose dextran (details of the animal feeding strategy are described in Figure 1A), this was done on the sixteenth day. Following a nine-hour gavage, the mice were euthanized and their samples were taken.

Plasma samples were collected, centrifuged for 10 min at 4°C at 10,000 *g*, and the supernatants were stored at −80°C for biochemical and ELISA analysis. The liver and colon tissues were extracted right away, and they were then cleaned in a phosphate-buffered saline solution. After that, the samples were stored for further analysis at −80°C.

### Biochemical and ELISA analysis of the serum and liver

ELISA kits were used to measure the liver levels of myeloperoxidase (MPO), interleukin-1β (IL-1β), tumor necrosis factor alpha (TNF-α), interleukin-10 (IL-10) interleukin-22 (IL-22) and the serum levels of lipopolysaccharide (LPS). Biochemical kits were utilized to determine the serum levels of alanine transaminase (ALT), aspartate transaminase (AST) and the liver level of triglyceride (TG). Mei Mian Biotechnology in Jiangsu, China and Nanjing Jiancheng Co., Ltd. in Nanjing, China, were the providers of all the kits.

### Real-time quantitative PCR

The RNAeasy^™^ Animal RNA Isolation Kit with Spin Column (Beyotime Biotechnology, Shanghai, China) was utilized to extract total RNA from tissue samples. Using the HiScript^®^ II Q RT SuperMix for qPCR (Vazyme, Nanjing, China), cDNA was produced by PCR (42°C, 2 min; 50°C, 15 min; 85°C, 5 s). Using Taq Pro Universal SYBR qPCR Master Mix (Vazyme, Nanjing, China) and QuantStudio3 (Thermo Fisher Scientific, PA, USA), real-Time Quantitative PCR was performed using the following PCR settings: 95°C, 30 s; 40 cycles of (95°C, 5 s, 60°C, 10 s; 72°C, 20 s). The comparative Ct method (2^−ΔΔCt^) was utilized to calculate the levels of gene expression. For a quantitative assessment of RNA amplification, each cDNA sample was examined in duplicate. Supplementary Data 1 provides an overview of primers used.

### Staining procedures

As described (Wang et al., 2016), the liver samples were formalin-fixed and sectioned for hematoxylin–eosin (H&E) staining. Oil red O (Wang et al., 2016) was used to stain sections of frozen liver. All sections were scanned by Nikon Eclipse 80i microscope (Nikon, Kobe, Japan).

### 16S rRNA sequencing and bioinformatics

Each group had cecum fecal DNA samples collected for the 16S rRNA gene sequencing and analysis. Utilizing the Qiagen Stool DNA Extraction Kit, DNA was extracted and utilized at Novogene (Tianjin, China) for sequencing on an Illumina MiSeq instrument. Using Vsearch, the sequences that shared a similarity level of greater than 97% were clustered into operational taxonomic units (OTU). A USEARCH and VSEARCH-based pipeline was used to evaluate raw sequence data. Using QIIME, a PCoA plot was generated using the Bray-Curtis and UniFrac distances (Ramette, 2007). For the purpose of identifying differentiated taxa, the linear discriminant analysis (LDA) effect size (LEfSe) was then determined (Segata et al., 2011). The significance of the LEfSe differences between biological groups was assessed using the non-parametric factorial Kruskal-Wallis test and the Wilcoxon rank-sum test. 16S rRNA sequencing data have been deposited in the NCBI Sequence Read Archive (SRA) repository under accession number: PRJNA1038794.

### Metabolomics analysis of feces

Following the weighting of 25 mg of the sample, 500 μL of the extract solution (methanol: acetonitrile: water = 2: 2: 1, with an internal standard mixture that was isotopically labeled) was added. Following a 4-min homogenization at 35 Hz, the samples were sonicated for 5 min in an ice-water bath. Three cycles of homogenization and sonication were carried out. The samples were then centrifuged for 15 min at 4°C at 12,000 rpm after being incubated for 1 h at −40°C. For analysis, the resultant supernatant was transferred to a brand-new glass vial. An equal aliquot of the supernatants from each sample was combined to create the quality control (QC) sample. An Orbitrap MS mass spectrometer (Orbitrap MS, Thermo) was coupled to a UPLC BEH Amide column (2.1 mm × 100 mm, 1.7 μm) in a UHPLC system (Vanquish, Thermo Fisher Scientific) for the LC–MS/MS analysis (Wang et al., 2016). 25 mmol/L ammonium hydroxide and 25 mmol/L ammonium acetate in water (pH = 9.75) (A) and acetonitrile (B) made up the mobile phase. The injection volume was 2 μL, and the auto-sampler temperature was 4°C. Since it can get MS/MS spectra in information-dependent acquisition (IDA) mode and manage the acquisition software (Xcalibur, Thermo), the Orbitrap Exploris 120 mass spectrometer was utilized. The acquisition software continuously evaluates the whole scan MS spectrum in this mode. The following parameters were set for the ESI source: capillary temperature of 320°C, full MS resolution of 60,000, MS/MS resolution of 15,000, collision energy of 10/30/60 in NCE mode, sheath gas flow rate of 50 Arb, aux gas flow rate of 15 Arb, spray voltage of 3.8 kV (positive) or −3.4 kV (negative), respectively. Proteo Wizard was used to convert the raw data into the mzXML format. An internal software built with R and based on XCMS was then used to process the data for peak detection, extraction, alignment, and integration (Smith et al., 2006). Next, an MS2 was used for the annotation of metabolites. The database (BiotreeDB) cutoff for annotations was set at 0.3.

### Statistical analysis

GraphPad Prism 8 was a software program we utilized to analyze our data. Where appropriate, the Dunnett’s multiple comparison test and Unpaired two-tailed Student’s *t*-test were employed after the ordinary one-way ANOVA. At *p* < 0.05, statistical significance was taken into account.

## Results

### *Bacillus coagulans* reduced histological damage of liver in ethanol-induced mice

We investigated the impact of *B. coagulans* on ethanol-induced ALD using the Lieber DeCarli diet with 5% ethanol. For 16 days, mice in the healthy control group were given the Lieber-DeCarli diet. Mice in the ethanol treatment group started eating a Lieber-DeCarli diet containing 5% ethanol on the fifth day, while mice in the *B. coagulans* treatment group started receiving *B. coagulans* by gavage every 2 days on the ninth day (Figure 1A). In comparison to the ethanol-induced group, liver lesions were recovered when *B. coagulans* was supplemented. This was shown by decreased ratio of liver to total body weight (Figure 1B), as well as a decreased serum ALT and AST levels (Figures 1C,D).

To investigate more about the function of *B. coagulans* in the histology of the liver tissue in mice given ethanol, we measured the hepatic triglycerides (TG) and stained for hepatic. It is evident that exposure to ethanol causes TG levels to rise greatly; nevertheless, the addition of *B. coagulans* caused TG levels to drop sharply (Figure 1E). Analysis of myeloperoxidase (MPO) activity showed that *B. coagulans* successfully reduced neutrophil infiltration compared to the ethanol-induced group (Figure 1F). In addition, alterations in the livers’ histological structure were assessed using H&E and Oil Red O staining (Figure 1G). Compared to the healthy control group, ethanol caused the cells to become denaturated and expanded, and the cytoplasm to fill with balloon-like fat vacuoles, which resulted in a significant accumulation of macrovesicular fat deposition in the liver. However, the ethanol-induced liver lesions were restored by *B. coagulans*, and the liver histology was similar with that of the healthy control group (Figure 1G). These results demonstrated that *B. coagulans* was a beneficial treatment for mice’s ethanol-induced liver lesions.

### *Bacillus coagulans* improved inflammatory response in ethanol-induced mice

**Figure 2:**
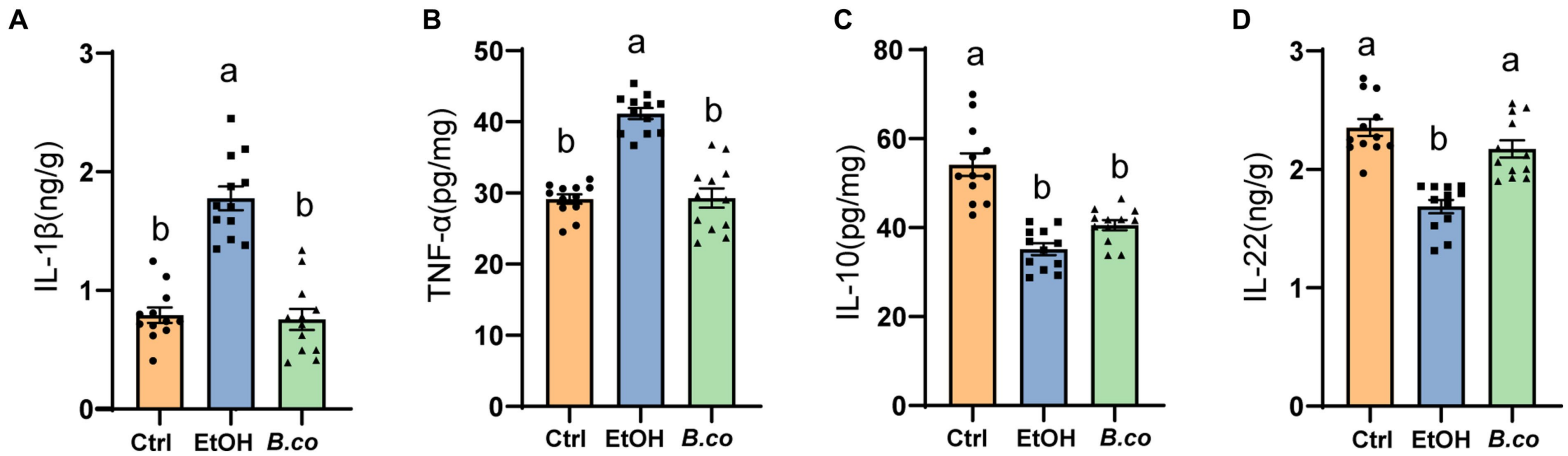
Effect of *B. coagulans* on regulation of immune markers in ethanol-induced mice. Protein levels of cytokines, including **(A)** IL-1β, **(B)** TNF-α, **(C)** IL-10, and **(D)** IL-22 of liver, were analyzed by ELISA. Data are shown as means ± SEM (*n* = 12 per group). Data with different superscript letters (a, b, and c) are significantly different (*p* < 0.05) according to one-way ANOVA followed by Tukey’s test. Ctrl, healthy control group; EtOH, ethanol-induced group; *B. co*, supplementation of *B. coagulans* group.

We further evaluate *B. coagulans* supplementation’s impact on ALD mice’s inflammatory responses. It has been demonstrated that pro-and anti-inflammatory factors influence the immune response in ALD. Overall, compared to the ethanol-induced group, the liver of the *B. coagulans* group exhibited significantly lower levels of the proinflammatory cytokines IL-1β (Figure 2A) and TNF-α (Figure 2B). In addition, compared to the ethanol-induced group, the *B. coagulans* treatment raised the levels of the anti-inflammatory cytokine IL-10, but this increase was not significant (Figure 2C). Similarly, ethanol dramatically decreased the amount of the cytokine IL-22 in the mice’s liver (Figure 2D); yet, *B. coagulans* supplementation further enhanced this reduction. In conclusion, the cytokines were successfully modulated by the supplement of *B. coagulans*.

### *Bacillus coagulans* restored the barrier function in ethanol-induced mice

**Figure 3:**
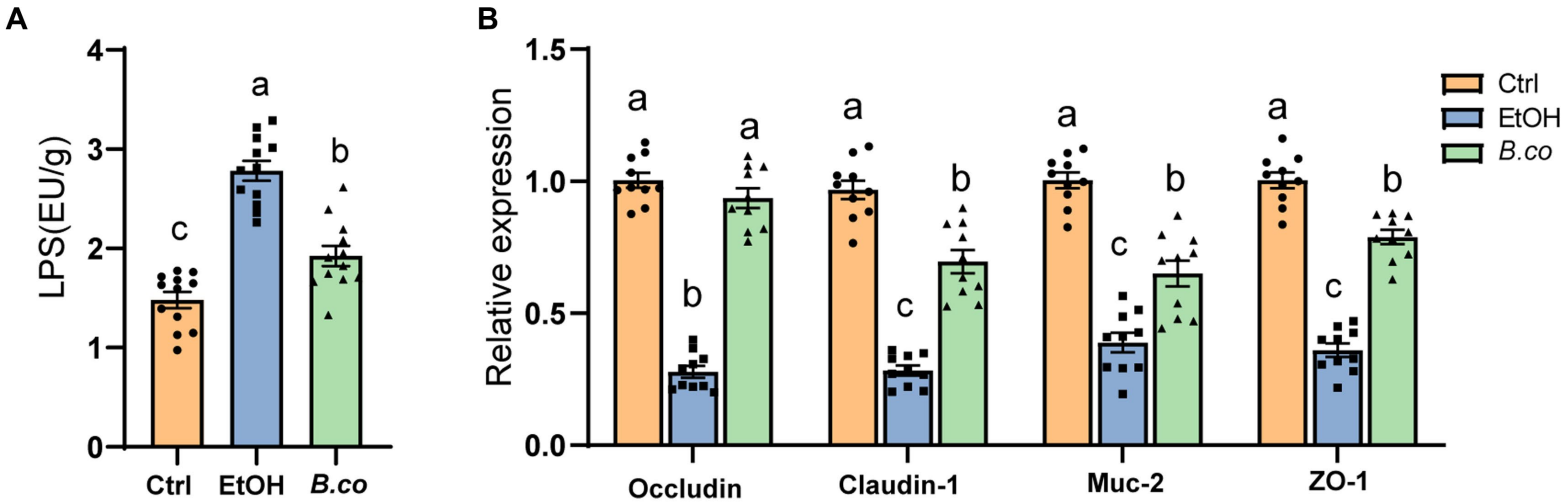
*B. coagulans* restored the barrier function in ethanol-induced mice. **(A)** Serum lipopolysaccharide concentrations (*n* = 12 per group). **(B)** The mRNA levels of mucin protein Muc2 and tight junction proteins (Occludin, Claudin-1 and ZO-1) were detected by quantitative RT-qPCR analysis in colon (*n* = 10 per group). Data are shown as means ± SEM. Data with different superscript letters (a, b, and c) are significantly different (*p* < 0.05) according to one-way ANOVA followed by Tukey’s test. Ctrl, healthy control group; EtOH, ethanol-induced group; *B. co*, supplementation of *B. coagulans* group.

It has been observed that mice given alcohol exhibit reduced mucosal barrier and a disturbs intestinal mucus layer (Grander et al., 2018). Therefore, in order to investigate potential processes by which *B. coagulans* offered protection against ALD, serum LPS levels have been examined. It is possible that ethanol actually causes disruption of the intestinal barrier in mice, which leads to the leakage of LPS, as seen by the significantly greater levels of LPS in the serum of the ethanol-induced group compared to the healthy control group (Figure 3A). Notably, compared to the ethanol-induced group, mice treated with *B. coagulans* had reduced levels of LPS (Figure 3A). This suggests that in an ethanol-induced model of ALD, *B. coagulans* supplementation would be able to enhance the intestinal barrier.

In order to learn more about *B. coagulans’* function in maintaining the integrity of the intestinal barrier in the colon, the colonic tight junction protein was examined. The tight junction proteins Occludin, Claudin-1, and ZO-1 as well as the mucin protein Muc-2 were transcriptionally downregulated in the ethanol-induced mice when compared to the healthy control group (Figure 3B). By comparison with the ethanol-induced group, *B. coagulans* markedly upregulated the expression of Occludin, Claudin-1, ZO-1 and Muc-2 (Figure 3B). All things considered, these findings suggest that *B. coagulans* increases the expression of mucin and tight junction (TJ) proteins, improving intestinal integrity and barrier function in mice with ethanol-induced damage.

### *Bacillus coagulans* supplementation regulated the composition and SCFAs production of gut microbiota

**Figure 4:**
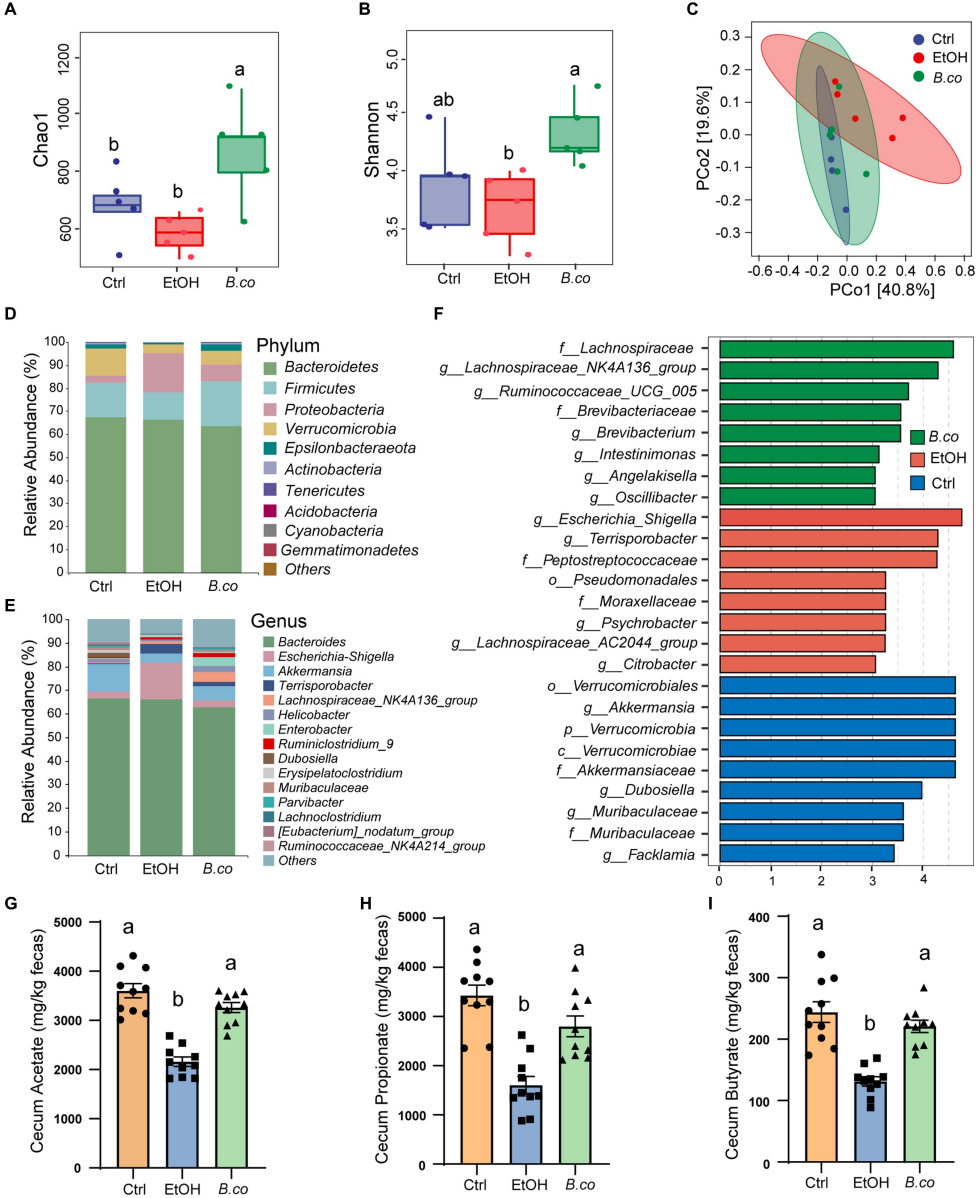
*B. coagulans* regulated the composition of gut microbiota. **(A,B)** Alpha-diversity represented by the Chao1 **(A)** and Shannon **(B)** indices. **(C)** Principal coordinates analysis of Bray-Curtis distance. **(D,E)** The relative abundance of fecal bacterial phylum **(D)**, and genus **(E)**. **(F)** Analysis of differences in the microbial taxa by LEfSe (current linear discriminant analysis (LDA) threshold is 3) in different groups. Concentrations of fecal acetate **(G)**, propionate **(H)**, and butyrate **(I)**. (**A–F**: *n* = 5 per group; **G–I**: *n* = 10 per group) Data are shown as means ± SEM. Data with different superscript letters (a, b, and c) are significantly different (*p* < 0.05) according to one-way ANOVA followed by Tukey’s test. Ctrl, healthy control group; EtOH, ethanol-induced group; *B. co*, supplementation of *B. coagulans* group.

We further investigate the effect of *B. coagulans* on the composition of the gut microbiota in mice treated with ethanol by employing 16S rRNA gene sequencing of fecal samples. Treatment with *B. coagulans* significantly improved alpha diversity in ALD mice as evidenced by increases in the Chao1 and Shannon index. This suggests that the *B. coagulans* treatment greatly increased the richness and diversity of the microbial community (Figures 4A,B). Principal coordinates analysis (PCoA) based on the Bray-Curtis distance showed that the gut microbiota structure differed between the mice given ethanol and the healthy control group (Figure 4C). Additionally, the gut microbiota structure of the mice treated with *B. coagulans* differed noticeably from that of the mice given ethanol, indicating that the *B. coagulans* supplement had a significant impact on the gut microbiota structure of the ethanol-induced mice. In addition, the community clustering with *B. coagulans* supplementation showed a significant similarity to the healthy group compared to the ethanol-induced group (Figure 4C).

The gut microbiotas of all groups displayed comparable phylogenetic structures, despite considerable differences in the proportional abundances of each component. The gut microbiota was primarily composed of *Bacteroidetes*, *Firmicutes*, *Proteobacteria*, *Verrucomicrobia* and *Epsilonbacteraeota* (Figure 4D). When compared to the healthy control group, the ethanol-induced group showed a notable decrease in *Firmicutes*, *Verrucomicrobia* and *Epsilonbacteraeota*, while *Proteobacteria* showed a significant increase. *Firmicutes*, *Verrucomicrobia* and *Epsilonbacteraeota* considerably increased and *Proteobacteria* significantly decreased following *B. coagulans* treatment. In each group, the most common genera were *Bacteroides*, *Escherichia-Shigella*, *Akkermansia*, *Terrisporobacter* and *Lachnospiraceae* (Figure 4E). The ethanol-induced group showed a noticeable decrease in *Akkermansia* abundance and a large increase in *Escherichia-Shigella* abundance when compared to the healthy control group. Additionally, following *B. coagulans* treatment, the gut microbiota’s composition was brought back to that of the healthy control group, showing notable improvements in the *Akkermansia* and *Lachnospiraceae_NK4A136_group* relative to the ethanol-induced group. The microbial taxa that were differentially abundant in ethanol-induced mice in response to *B. coagulans* supplement could be found using LEfSe analysis (Figure 4F). Pathogenic bacteria, including *Escherichia-Shigella*, *Terrisporobacter*, and *Peptostreptococcaceae*, were only found to be enriched in the ethanol treatment group. Conversely, the *B. coagulans* treatment enhanced *Lachnospiraceae_NK4A136_group*, *Ruminococcaceae_UCG-005* and *Oscillibacter*, whereas the healthy control group exhibited the highest abundance of *Akkermansia* and *Muribaculaceae*. These results revealed that one of the processes supporting the mice’s health in this study was the enrichment of beneficial bacteria in the *B. coagulans* treatment group. Unfortunately, 16S rRNA gene sequencing did not identify *B. coagulans* in the feces of the *B. coagulans* treatment group. This is likely due to the bacterium’s limited colonization capacity.

To investigate the impact of *B. coagulans* supplementation on SCFAs generation in greater detail, we measured the fecal concentrations of acetate, propionate, and butyrate. All three SCFAs were significantly lowered by ethanol. Overall, the *B. coagulans* treatment group had a noticeably higher concentration of SFCAs (Figures 4G–I). These results suggest that *B. coagulans* was associated with higher SFCAs in the gut compared to the ethanol group.

### *Bacillus coagulans* supplementation effected the metabolomic profiles

**Figure 5:**
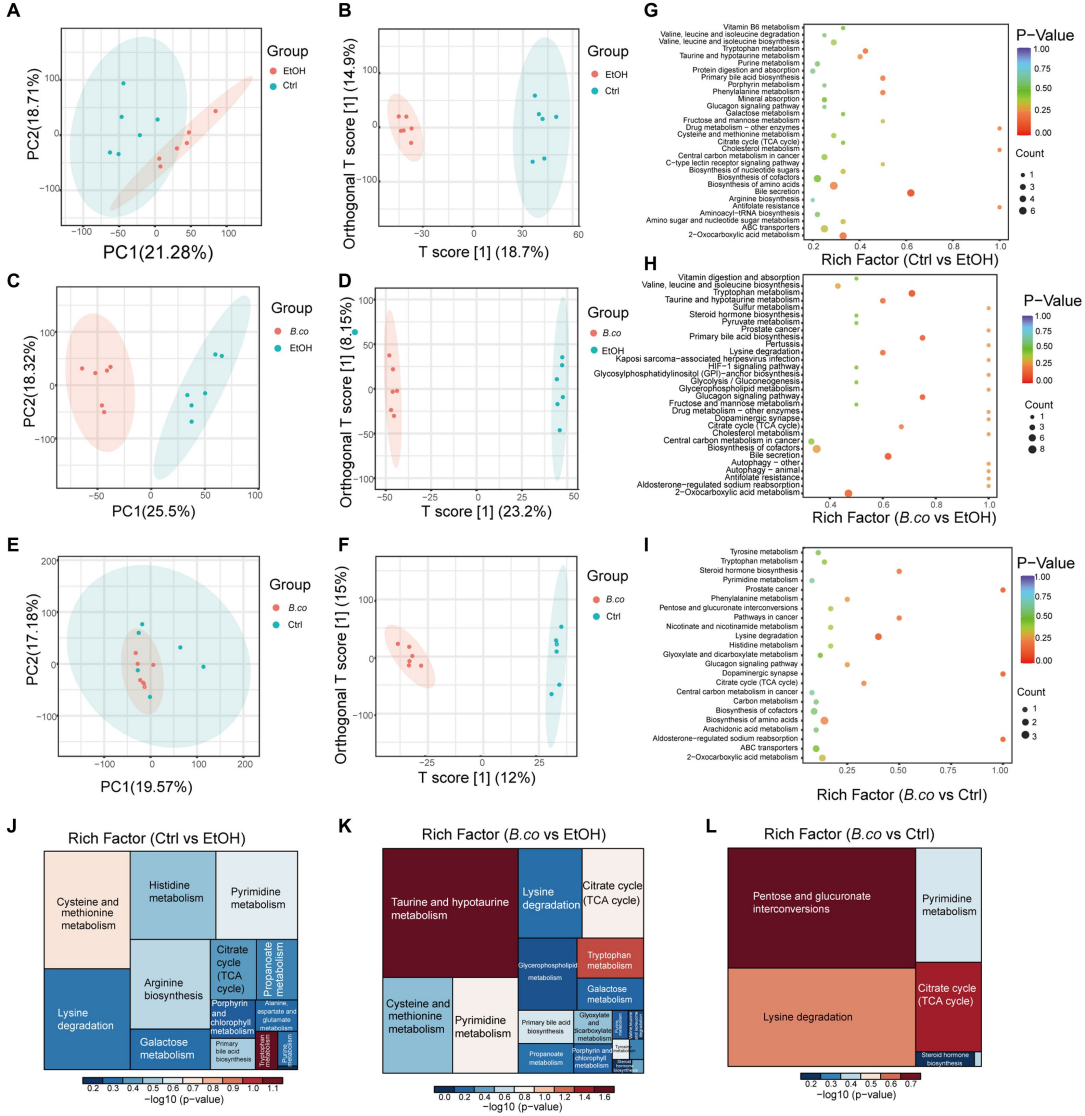
Metabolic patterns in the experiment groups. **(A)** PCA analysis between healthy control group and ethanol-induced group. **(B)** Clustering analysis of OPLS-DA in the healthy control group and ethanol-induced group. **(C)** PCA analysis between *B. coagulans*-treated group and ethanol-induced group. **(D)** Clustering analysis of OPLS-DA in the *B. coagulans*-treated group and ethanol-induced group. **(E)** PCA analysis between *B. coagulans*-treated group and healthy control group. **(F)** Clustering analysis of OPLS-DA in the *B. coagulans*-treated group and healthy control group. **(G)** The KEGG pathways in the healthy control group and ethanol-induced group. **(H)** The KEGG pathways in the *B. coagulans*-treated group and ethanol-induced group. **(I)** The KEGG pathways in the *B. coagulans*-treated group and healthy control group. **(J)** Pathway analysis for group ethanol-induced vs. healthy control. **(K)** Pathway analysis for group *B. coagulans*-treated vs. ethanol-induced. **(L)** Pathway analysis for group *B. coagulans*-treated vs. healthy control. Ctrl, healthy control group; EtOH, ethanol-induced group; *B. co*, supplementation of *B. coagulans* group.

The metabolomics data of the fecal were shown in Figure 5. PC1 accounted for 21.28% and PC2 accounted for 18.71% of the ethanol-induced group’s total compared to the healthy control group (Figure 5A). Between the *B. coagulans* treatment group and the ethanol-induced group, the PC1 accounted for 25.50% and the PC2 for 18.32% (Figure 5C). In the comparison between the *B. coagulans* treatment group and the healthy control group, PCl accounted for 19.57% and PC2 for 17.18% (Figure 5E). Figures 5B,D,F, respectively, shows the outcomes of the orthogonal partial least-squares discriminant analysis (OPLS-DA) of the metabolomic between the ethanol-induced, the *B. coagulans* treatment group, and the healthy control group. After being treated with ethanol or *B. coagulans*, the metabolites changed significantly in comparison to the group of healthy controls. Fecal metabolites were annotated and found to be highly enriched in the KEGG pathways associated with “Bile metabolism,” “Tryptophan metabolism,” “2-Oxocarboxylic acid metabolism,” “Primary bile acid biosynthesis,” and “Phenylalanine metabolism” between the ethanol-induced and healthy control groups (Figure 5G). Similarly, the KEGG pathways associated with “Tryptophan metabolism,” “Bile secretion,” “Primary bile acid biosynthesis,” “Glucagon signaling pathway,” and “2-Oxocarboxylic acid metabolism” were highly enriched between the *B. coagulans* treatment and the healthy control groups (Figure 5H). Moreover, the KEGG pathways associated with “Lysine degradation,” “Dopaminergic synapse,” “Aldosterone-regulated sodium reabsorption,” “Prostate cancer,” and “Steroid hormone biosynthesis” were highly enriched between the *B. coagulans* treatment and the ethanol-induced groups (Figure 5I). We can further identify the metabolic pathways associated with the three groups of differentially expressed metabolites by conducting a thorough analysis of differential metabolite pathways, which includes enrichment and topological analyses. This will allow us to identify the key pathways that have the highest correlation with metabolite differences (Figures 5J–L). The findings revealed that the three groups’ differential metabolites showed significant enrichment in several metabolic pathways, including tryptophan metabolism, citrate cycle (TCA cycle), taurine and hypotaurine metabolism, pentose and glucuronate interconversions, lysine degradation, and others.

**Figure 6:**
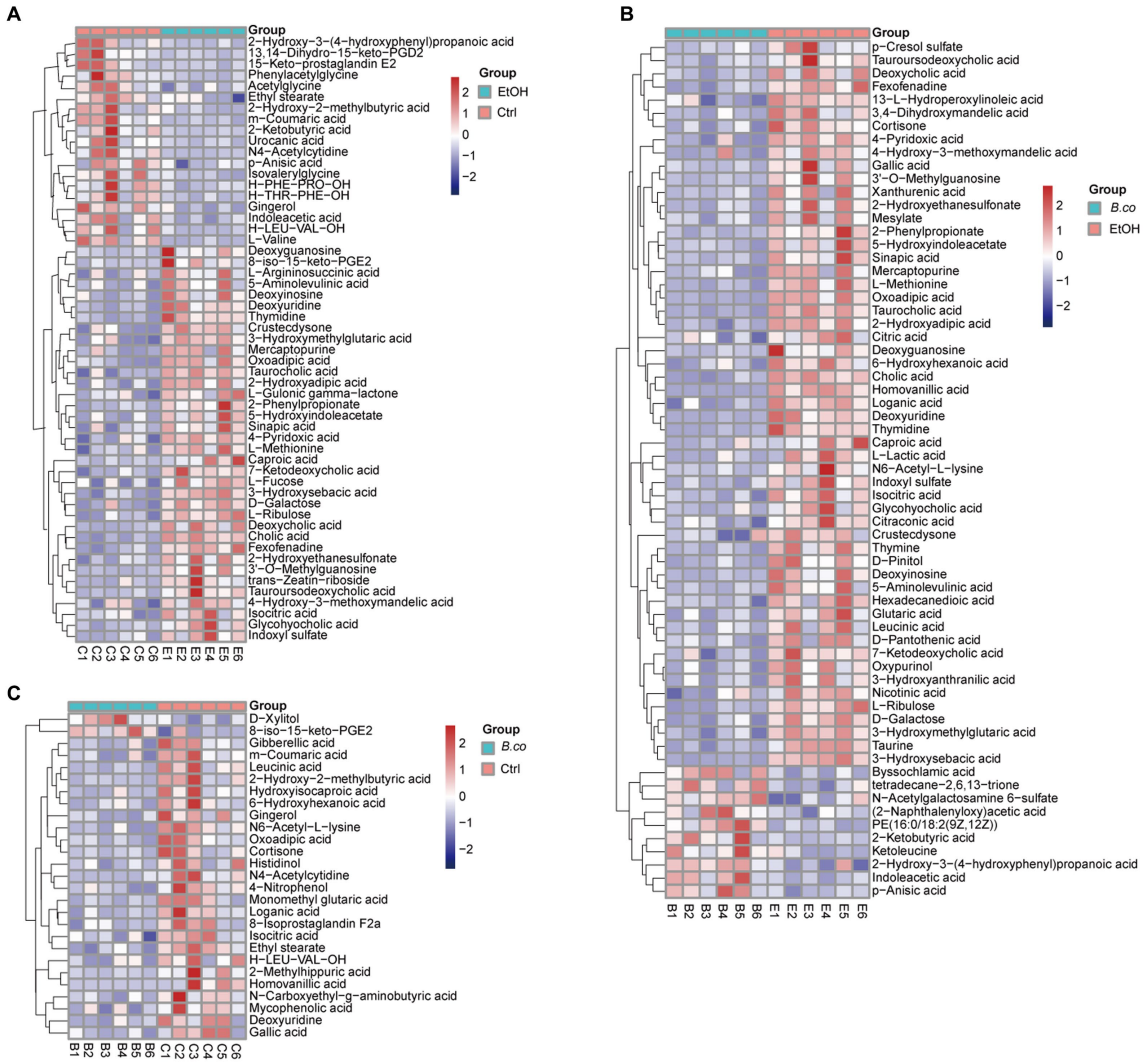
The heatmap of hierarchical clustering analysis different metabolites. **(A)** The heatmap of different metabolites for group healthy control vs. ethanol-induced. **(B)** The heatmap of different metabolites for group *B. coagulans*-treated vs. ethanol-induced. **(C)** The heatmap of different metabolites for group *B. coagulans*-treated vs. healthy control. Ctrl, healthy control group; EtOH, ethanol-induced group; *B. co*, supplementation of *B. coagulans* group.

The different metabolites detail was shown in Figures 6A–C. When ethanol was administered, the detail level of several metabolites, such as 3’-O-Methylguanosine, cholic acid, thymidine, and caproic acid, increased significantly in comparison to the healthy control group. Additionally, there was a noticeable decrease in the abundance of 2-Ketobutyric acid, N4-Acetylcytidine, phenylacetylglycine, and L-Valine (Figure 6A). Furthermore, after being treated with *B. coagulans*, the abundance of PE (9z,12Z), 2-ketobutyric acid, ketoleucine, and indoleacetic acid was significantly increased by hierarchical clustering analysis, while that of homovanillic acid, thymidine, deoxyuridine, 3’-O-Methylguanosine, cholic acid, and 3-hydroxysebacic acid was obviously decrease in comparison to the ethanol-induced group (Figure 6B). We discovered that while the levels of D-Xylitol and 8-iso-15-keto-PGE2 were enriched between the *B. coagulans* treatment and the healthy control groups, the levels of homovanillic acid, N4-Acetylcytidine, 2-Hydroxy-2-methylbutyric acid, and leucinic acid were clearly decreased (Figure 6C). These findings suggest to a possible therapeutic function for homovanillic acid and indoleacetic acid, in the treatment of ethanol-induced ALD, and a possible correlation between 2-Ketobutyric acid, 3’-O-Methylguanosine, and cholic acid.

## Discussion

The most common feature of ALD is hepatic steatosis, primarily brought on by the intracellular accumulation of triglycerides in hepatocytes. The accumulation of these triglycerides as micro-and macrovesicles has the potential to upset the cell’s structural integrity (Seitz et al., 2018). The course of ALD is intimately linked to the development of inflammation, the ensuing hepatocyte destruction, and a process called ballooning (Teschke, 2018). Intestinal dysbiosis and compromised intestinal barrier integrity are well-known to be factors in inflammatory liver damage (Dukić et al., 2023; Wu et al., 2023). These kinds of events are also observed in cases of long-term alcohol abuse. Numerous studies have been conducted on the significance of the gut microbiota in maintaining organismal homeostasis and its possible therapeutic uses for ALD (Gu et al., 2019; Dukić et al., 2023). In order to confirm our theory that supplement *B. coagulans* was related to the ethanol-induced ALD, we employed C57 mice in breeding experiments. The study’s findings unmistakably showed that including *B. coagulans* in the diet significantly reduced the symptoms and severity of ethanol-induced ALD in mice. Evidence for this phenomenon was provided by the ethanol-induced model of ALD, which improved liver cells denatured and enlarged, balloon-like fat vacuoles, macrovesicular fat accumulation, hepatomegaly, TG accumulation, intestinal microbiota disorders, inflammation disorders, reduced SCFAs levels, reduced tight junction protein levels, and metabolic disorders. It is unclear exactly how *B. coagulans* supplementation regulates metabolites or directly stimulates the expression of TJs and Muc2 proteins, but it was found to dramatically increase the expression of these ethanol-degraded proteins, bringing them back to levels similar to those in the healthy group. These results imply that *B. coagulans* supplementation markedly enhanced the epithelium’s integrity in mice given ethanol. When considered collectively, these findings suggest the potential therapeutic use of *B. coagulans* in treating ALD by supporting its ability to restore epithelial integrity in ethanol-induced mice models.

Alcohol consumption has been associated with alterations in the gut microbiota in humans, and dysbiosis is thought to be a major factor in the development of ALD (Macnaughtan and Jalan, 2015; Grander et al., 2018; Hsu and Schnabl, 2023). Patients with ALD exhibit an increase in *Proteobacteria* (Mutlu et al., 2012). Recent research has shown that the dysbiotic gut microbiota of individuals with ASH increases their susceptibility to ALD (Llopis et al., 2016). Reduced *A. muciniphila* presence is a hallmark of the dysbiosis seen in ASH patients and mice fed the Lieber-DeCarli ethanol diet (Grander et al., 2018). Alternative experimental that include ALD research have shown somewhat contradictory results in the field of puzzles, suggesting an increase in *A. muciniphila* populations after exposure to ethanol (Yan et al., 2011; Hartmann et al., 2013; Wang et al., 2016). It is still unclear how exactly ethanol reduces the amount of *A. muciniphila* in the environment. Despite the fact that ethanol did not impede *A. muciniphila*’s growth *in vitro* (Grander et al., 2018), it is possible that other (such as indirect) mechanisms contribute to the microbial community’s depletion of ethanol. We now show that ethanol reduced the amount of *A. muciniphila* in the mouse gut, but that this effect was countered by *B. coagulans* supplementation. One limitation of our study is that we were unable to determine the exact mechanism by which *B. coagulans* acts in the gut to increase the amount of *A. muciniphila*.

According to our hypothesis, *B. coagulans* may play a part in regulating the gut microbiota, acting as an antimicrobial against pathogenic bacteria, and promoting the restoration of the intestinal barrier (Abdhul et al., 2015; Mu and Cong, 2019). This theory seems to be further supported by the following decrease in potentially pathogenic bacteria, such as *Escherichia-Shigella*, *Terrisporobacter* and *Peptostreptococcaceae*, and the simultaneous increase in beneficial bacteria, such as *Lachnospiraceae_NK4A136_group*, *Ruminococcaceae_UCG-005* and *Oscillibacter*. Microbiota-produced metabolites, like short-chain fatty acids (SCFAs), have the potential to reduce inflammation.

Metabolites produced by the microbiota, such as short-chain fatty acids (SCFAs), may exert anti-inflammatory effects, modulate intestinal immune activity, and enhance epithelial barrier function (Vernocchi et al., 2016; Cheng et al., 2022). Our results are consistent with a previous study (Liu et al., 2022) that showed supplementing with *B. coagulans* can increase levels of SCFAs. It was shown that *B. coagulans* treatments markedly increased the levels of SCFAs, which may be linked to better intestinal barrier integrity, improved liver histology, and less severe illness in ethanol-induced mice.

A number of mechanisms and processes, including the production and release of cytokines and chemokines by different cell types, including hepatocytes, mediate the course of ALD (Wu et al., 2023). Both ALD patients and chronic ethanol-fed animals have been shown to have elevated levels of several cytokines, including TNF-α and IL-1β. The majority of these cytokines have two roles in the pathophysiology of ALD (Nagy et al., 2016; Wang et al., 2021). As a vital proinflammatory cytokine in ALD, TNF-α is essential (Nagy et al., 2016; Wang et al., 2021).

Long-term ethanol exposure causes lipopolysaccharide (LPS) in the gut to translocate, activating kupffer cells (KCs) via Toll-like receptor 4 (TLR4). This leads to increased synthesis of proinflammatory cytokines, such as TNF-α and IL-1, which promote hepatocyte dysfunction and programmed cell death (PCD). Additionally, it activates hepatic stellate cells, which produce extracellular matrix (ECM) proteins that cause the development of cirrhosis and fibrosis (Tilg et al., 2016). Interestingly, mice defective in several components of the IL-1 pathway, *tnf-α* knockout mice, and mice treated with IL-1 receptor antagonist to neutralize IL-1 activity show protection against ethanol-induced liver injury (Mathews and Gao, 2013). The potential application of *B. coagulans* in the treatment of ALD is supported by its ability to regulate the expression levels of TNF-α under inflammatory conditions, as suggested by previous studies (Shinde et al., 2019, 2020; Liu et al., 2022). This suggests that the primary immunomodulatory capability of the bacteria may be responsible for its anti-inflammatory effect. However, when combined with data on the intestinal barrier, immune response, and tissue damage, we discovered that *B. coagulans* by itself was unable to completely restore the injured mice. Additionally, it has been reported that *B. coagulans* combining with prebiotics enhanced its probiotic characteristics (Shinde et al., 2019, 2020; Liu et al., 2022). Therefore, in future studies, it may be possible to treat ALD mice using *B. coagulans* in Combination with prebiotics.

One important clinical indicator of ALD patients is impaired epithelial barrier due to disruption of intestinal epithelial tight junctions (TJs), which creates intracellular spaces between adjacent epithelial cells and allows pathogens to pass through them (Dukić et al., 2023; Wu et al., 2023). Consistent with our findings, previous research has demonstrated that *B. coagulans* supplementation can upregulate the expression of TJs and mucin protein (Shinde et al., 2019, 2020; Liu et al., 2022). To understand the processes behind *B. coagulans*-mediated mitigation of ethanol-induced gastrointestinal and hepatic injuries, we further analyzed intestinal metabolites. A greater number of genes related to amino acid metabolism were present in the microbiota that *B. coagulans* altered. It suggests that the metabolites 2-Ketobutyric acid, 3’-O-Methylguanosine, cholic acid, homovanillic acid, and indoleacetic acid are related to the treatment of ethanol-induced ALD mice by *B. coagulans*. We found a decrease in homovanillic acid levels in particular. This decrease has been associated to a decrease in metabolic stress (Luo et al., 2022), which could account for the decline in homovanillic acid levels we saw after *B. coagulans* supplementation in mice with ethanol-induced ALD. Some microbes synthesize a tryptophan metabolite known indole, which can activate the aryl hydrocarbon receptor (AhR) (Hendrikx et al., 2019). This enhances the synthesis of mucus and antimicrobial peptides, as well as the function of the intestinal barrier by promoting the expression of IL-22 (Zheng et al., 2008). It has been demonstrated that ALD (Wrzosek et al., 2021), metabolic syndrome (Natividad et al., 2018; Hendrikx et al., 2019), and inflammatory bowel disease (Lamas et al., 2016) can all be improved by AhR activation. Furthermore, IL-22 is involved in protecting the ethanol-induced injury. Recombinant IL-22 protein injection stimulates hepatic STAT3 and protects mice against hepatic oxidative stress and hepatocyte injury in the Gao-binge mouse model of ALD. Recombinant IL-22 fusion protein (F-652) was found to significantly improve clinical scores and decrease markers of liver injury in patients with ALD during a human phase II study (Ki et al., 2010; Arab et al., 2020). By modulating intestinal bacteria’ capacity to produce indole derivatives, *B. coagulans* may enhance the expression of IL-22, thereby restoring the intestinal barrier, and treating ALD in mice. This is supported by the up-regulation of tryptophan metabolism and indoleacetic acid following *B. coagulans* supplementation in this study.

Probiotics and ALD have been linked, and this link has received a lot of attention and validation. In the present study, mice were given ethanol to cause ALD, and *B. coagulans* was then added as a therapeutic supplement. We showed that supplementation *B. coagulans* considerably increased the richness and diversity of the gut microbiota community. Additionally, probiotic bacteria that are known to produce SCFAs were more abundant (Park et al., 2021). Enriched in the *B. coagulans* group are *Akkermansia*, *Lachnospiraceae*, *Ruminococcaceae* and *Oscillibacter*, which have been shown to be linked to the synthesis of SCFAs and have strong probiotic qualities (Martin-Gallausiaux et al., 2021). Consequently, *B. coagulans* may influence the probiotic gut microbiota’s ability to produce SCFAs either directly or indirectly, which would be advantageous for the control of ALD (Martin-Gallausiaux et al., 2021). These findings supported our hypothesis that *B. coagulans* could treat ALD mice by restoring the intestinal barrier through regulation of the gut microbiota, g production of SCFAs or indole derivatives, and then upregulating the expression of cytokines like IL-22.

## Data availability statement

The data presented in the study are deposited in the NCBI Sequence Read Archive (SRA) repository, accession number PRJNA1038794.

## Ethics statement

The animal study was approved by Institutional Animal Care and Use Committee regulations at the Huazhong Agricultural University. The study was conducted in accordance with the local legislation and institutional requirements.

## Author contributions

ZL: Data curation, Formal analysis, Funding acquisition, Writing – original draft, Writing – review & editing. TL: Data curation, Investigation, Methodology, Writing – original draft. ZZ: Conceptualization, Project administration, Writing – original draft. YF: Resources, Software, Writing – review & editing.
